# Extending the reach of palliative care–a double–edged sword: a qualitative study of clinicians’ experiences of delivering telehealth in Australia and Aotearoa New Zealand

**DOI:** 10.1186/s12904-025-01932-x

**Published:** 2025-11-13

**Authors:** Aileen Collier, Sinchana Appachoo, Rosemary Frey, Vicki Jones, Jacqueline Birtwistle, Matthew Allsop, Katherine Bloomfield

**Affiliations:** 1 Research Centre for Palliative Care, Death and Dying (RePaDD), Flinders University, Adelaide, South Australia Australia; 2 Faculty of Health and Medical Sciences, The University of Auckland, Auckland, Aotearoa, New Zealand; 3 Academic Unit of Palliative Care, Leeds Institute of Health Sciences, University of Leeds, Worsley Building, Leeds, UK

**Keywords:** Palliative care, Telehealth, Qualitative research, Health equity

## Abstract

**Background:**

Telehealth palliative care delivery has been shown to be feasible and acceptable for patients, families and clinicians across a number of contexts. However, the rapid implementation of telehealth during the pandemic exposed both challenges and opportunities for optimising telehealth integration in palliative care. This study capitalised on clinicians' experience to better understand the complexities of telehealth and palliative care with a focus on access and equity.

**Methods:**

We deployed a qualitative interview study exploring Australia and Aotearoa New Zealand (NZ) clinicians’ perspectives of telehealth with a focus on underserved palliative care populations. The study was underpinned by applied critical realism evaluation. Data were analysed through an equity lens using Srivastava and Hopwood’s framework, applying critical realist evaluation questions: what are these data telling us about what works, for whom, in which contexts and with what outcomes?

**Results:**

We recruited a total of twenty-two participants [doctors (*n* = 7) nurses (*n* = 11) allied health (*n* = 4)]. Fifteen participants were based in Aotearoa NZ and seven in Australia. Analysis resulted in the following four key themes: Extending the reach of palliative care; Underserved groups – the thin line of equity and access; Patient safety and quality- the complexities of clinical work; Tele-care, connection and creativity.

**Conclusions:**

Our findings show that although the adoption of telehealth can extend the reach of palliative care, there is, at the same time, the potential to further marginalise those for whom palliative care is already inaccessible. Support and education for clinicians are needed to ensure safe and high-quality telehealth, as well as organisational guidelines and structures to optimise the use of telehealth. There is a need for further research using ethnographic, participatory as well as other qualitative and quantitative methods to identify approaches that optimise access to palliative care and telehealth for underserved groups. This should include the perspectives of patients and families themselves.

## Background

For the purposes of this manuscript, we define telehealth as “the use of information and communication technologies to deliver health care when patients and care providers are not in the same physical location” [1]. Prior to the COVID-19 pandemic, the role of telehealth in palliative care remained under investigation. Known benefits of telehealth identified in the palliative care context included convenience, rapid access and reduced travel time [2, 3]. Telehealth delivery in palliative care was also known to be generally feasible and acceptable for patients and clinicians across several contexts [3, 4]. However, there was accelerated deployment of telehealth during the COVID-19 pandemic, providing a means for patients to continue to receive care even if they or healthcare professionals were quarantined [5]. This swift telehealth deployment also highlighted the importance of ensuring existing inequities were not further exacerbated, ensuring a focus on barriers to telehealth uptake amongst disadvantaged groups [6]. An abundance of evidence indicates that higher health and digital literacy, higher income, flexible work arrangements, from urban areas, and English-speaking ability [7] are linked to successful access to telehealth services [8–10]. Thus, there is a need to better understand the appropriateness, acceptability and effectiveness of telehealth palliative care for underserved populations, including, but not limited to older people [11], those with cognitive impairment, people of diverse cultural backgrounds [12], First Nations people and those living in rural and remote settings. The rapid and massive deployment of telehealth for people requiring palliative care in Australia and Aotearoa (NZ) during COVID-19 restrictions meant that clinicians enacted virtual treatment and care with and for patients and families that they previously would have performed in person. This study capitalised on the opportunity to better understand the complexities of telehealth and palliative care by exploring clinician experiences with a focus on access and equity.

### Aotearoa NZ and Australia context

Australia and Aotearoa NZ share a collegial palliative care relationship through for example, the professional organisation Australia New Zealand Society of Palliative Medicine. Further, both countries have Indigenous populations who were colonised. However, their health systems are very different. Australia’s specialist palliative care services are largely embedded within the public health system whereas Aotearoa’s specialist palliative care model is based on a hospice model partly funded by charitable donations.

In Aotearoa NZ, primary and specialist palliative care services are delivered in hospitals, aged residential care, private homes and hospices [13]. However, there have been challenges to the delivery of this care for a number of sectors of the community. For example, rural patients are on average demographically older than urban communities and often have more underlying health conditions and fewer economic resources leading to reduced access [14, 15]. In Aotearoa NZ, the health of Indigenous Māori is a right guaranteed by Te Tiriti o Waitangi, The Treaty of Waitangi [16]. However, as a group, Māori have experienced inequities in access to healthcare including palliative and end-of-life care [17]. With regard to technology, few tools have been developed to meet the needs of Māori communities. Furthermore, there is evidence of a lack of understanding of the social context in which those technologies were to be deployed. The result of this failure could further worsen health outcomes [18]. In Australia, a similar picture exists. Australia too provides both primary and specialist care. However, models of palliative care provision differ across each state and territory, with each having specified an approach [19]. The uptake of telehealth has been slow, fragmented and ad hoc across rural areas [20]. Unsurprisingly, health outcomes for rural patients are generally worse than their urban counterparts [21]. Likewise in remote Australian First Nations Communities, the burden of disease is high [22]. The acceptability of telehealth services depends upon culturally safe healthcare delivery [23], but there are known staff resource constraints for small clinics that can affect their capacity to deliver telehealth appointments [24].

## Methods

Our qualitative interview study was informed by an applied critical realism evaluation approach. That is, an epistemological space between positivism and relativism [25]. We sought to understand the mechanisms of telehealth through an equity lens drawing from clinicians’ (doctors, nurses, allied health staff) experiences during and prior to the pandemic and at the same time recognising mechanisms are transformed and reproduced by humans in interaction with other systems and actors. We leveraged relationships across countries to recruit diverse clinicians from a range of contexts. Ethics approval was sought and granted by the University of Auckland Human Participants Ethics Committee, no UAHPEC22090. We have applied the standards for reporting qualitative research criteria [26].

### Recruitment & data collection

We purposively recruited clinicians from Australia and Aotearoa NZ through the four lead palliative care organisations in NZ (Palliative Care Nurses NZ, Hospice NZ, Hospital Palliative Care NZ and The Australian & NZ Society of Palliative Medicine Inc. and associated organisations in Australia (Palliative Care Nurses Australia, Palliative Care Australia, The Australian & NZ Society of Palliative Medicine Inc.) We actively sought to recruit consenting clinicians working in services providing care to First Nations people and older adult health care practitioners as well as seeking variation across: (i) metropolitan and regional settings, (ii) professional discipline, (iii) telehealth experience (pre- and post-COVID-19 vs. post-COVID-19 only), and (iv) relevant telehealth clinical experiences within diverse clinical contexts. AC (an experienced qualitative researcher and palliative care nurse) oversaw data collection (March through October 2021) and was assisted by VJ (a medical educator, palliative care doctor and previously a GP) and a research assistant (SA) along with two Australia based clinical academic palliative care doctors. Before participating in interviews, all participants provided informed consent.

### Data analysis

Data analysis proceeded alongside data collection. Data were transcribed by a transcription service. We analysed data using Srivastava and Hopwood’s framework underpinned by applied critical realist evaluation questions [25] through an equity lens. That is, our analysis asked the question: what are these data telling us about the impact of telehealth on palliative care of underserved communities from the perspective of clinicians? We also asked the question: Whose voices are missing from our analysis? Interviews were recorded and transcribed verbatim. SA coded data and generated initial themes. These were continuously refined in regular meetings and via email with AC, VJ and KB (an experienced researcher and geriatrician). All (AC, VJ and KB) have direct experience in delivering palliative care via telehealth.

## Results

We interviewed a total of twenty-two participants [doctors (*n* = 7) nurses (*n* = 11) allied health (*n* = 4)] (See Table 1). Fifteen participants were based in Aotearoa NZ and seven in Australia. No participants identified as First Nations people. All interviews were online, and lasted between twenty-eight and sixty-nine minutes. All participants worked in specialist palliative care services except for four participants who worked in specialist older people services. Analysis resulted in four overarching themes: Extending the reach of palliative care; Underserved groups – the thin line of equity and access; Patient safety and quality- the complexities of clinical work; Tele-care, connection and creativity.

**Table 1:** Overview of participant characteristics

Category	Sub-category	Number of participants (*N* = 22)
Country setting	Australia Aotearoa New Zealand	7 15
Gender	Male Female	4 18
Work context	Metropolitan Non-metropolitan	17 5
Specialty	Specialist palliative care Older people services Paediatric specialist palliative care	16 4 2

**Professional roles of participants**		**Number of participants**
Nurse role (*n* = 11)	Nurse practitioner Clinical nurse specialist Nurse manager Registered Nurse	5 4 1 1
Medical role (*n* = 7)	Consultant/staff specialist	7
Allied health role (*n* = 4)	Physiotherapist Dietician Family support worker Pharmacist	1 1 1 1

### Extending the reach of palliative care

Participants, cognisant of the growing need for palliative care along with finite specialist resources, frequently spoke of using resources efficiently to maximise benefit for the communities they served. The doctor below compares service provision with other medical specialties to convey how telehealth complemented outpatient services, making better use of their time:

So we’re [specialist palliative care service] just trying to see how can we extend ourselves to extend our reach further. And face-to-face you can only do so much. We try and do as much work through the out-patient clinic as possible because, you know, patients coming in to see us probably and us going out to see patients just like any other specialty, we would encourage. (palliative care doctor, P2)

Participants of all disciplines and in both countries highlighted how travel to visit people at home, especially in rural areas, could take up a large portion of their time, limiting the number of people they could see. They conveyed how telehealth could enable more frequent touch points with patients and families promoting continuity of care as well as freeing up time:

If I have to go to Havelock or around to The Sounds, that’s probably three to four hours out of my eight-hour day for one or two people [patients]. So if you can do a visit once a fortnight, but actually telehealth in the middle, that gives them [patients and families] a lot better continuity and ongoing care. (nurse practitioner, P1)

Without the need to travel for home visits, clinicians conveyed how they were able to “see more patients and get more done”. They saw this as a way of being able to expand the reach of their services without additional funding. As another clinician noted:

There’s more and more patients in the community. And so there’s still the same budgets and so you can actually hold a bigger caseload if you are not visiting everybody. (palliative care doctor, P5)

The geographical challenges of providing specialist services meant that some participants reflected on the ‘greater good’ of limiting travel. For the doctor below there were sociopolitical as well as service provision reasons to deploy telehealth:

And you’ve gotta think about time, but also carbon, climate change issues, carbon credits and all that sort of stuff. So, a virtual appearance, you can see the patient, they can see you, that seems to be the way that things are going. And, you know, [I’ve] also heard about in Australia, outback Australia, we’re talking even vaster distances, you know, 1400 km to the closest clinic and that sort of thing. So, it’s just about, yeah, best use of time, best use of money, but also caring for the environment to some extent, as well. (palliative care doctor, P2)

Telehealth also enabled family members who were separated geographically to be present virtually and participate in a person’s care despite the separation of physical distance.

And it was actually quite effective in that some people who could never have come to the family meeting, for example, from Perth or from Western Australia, ah or from South Australia, they could actually beam into it and all be involved in this family meeting. So, it was surprisingly effective and everybody felt involved. (palliative care doctor, P2)

Virtual consultations meant that patients, family members and clinicians could come together in one ‘place’ in ways otherwise not feasible. This meant patient and family time was optimised as well as clinician’s time. Further, for some people this meant being able to direct their own care from their own home, promoting agency as the quote by a paediatric nurse below shows:

we had a young girl who was 15 who was dying of advanced cancer. And while she was still able to communicate with us, telehealth worked really well because she led that. She set it [telehealth meeting] up, she appreciated having that face-to-face contact. We’d dial in as a whole MDT, so there’d be the oncology nurse specialist, an oncologist, myself, the medical specialist and she [patient] loved it. It was great and she had more of a voice than she would have in person with that many people turning up at her house. (nurse specialist, P6)

In addition, telehealth provided specialist services the opportunity for flexible models of service delivery and workforce arrangements. The manager quoted below explained how specialist nurses providing virtual services could be based anywhere in Aotearoa NZ and were part of the team:

And now our clinical coordinators, who do the daily monitoring of patients and answering of phone calls and are both CNS’s (clinical nurse specialists) as well, now don’t live in Auckland. (hospice manager, P7)

### Underserved groups- the thin line of equity and access

This theme highlighted how, on the one hand, telehealth had the potential for people to be able to access palliative care who may otherwise not be able to. At the same time, however, telehealth had the potential to magnify already existing equity issues and/or have unintended consequences for some groups of people, including patients with cognitive impairment, children, people whose first language was not English, people with intellectual disability and First Nations people. The availability of telehealth consultations led clinicians to consciously reflect on the relative exclusion of rural patients from specialist palliative care services and evaluate the potential that telehealth had to bridge this disparity in access. This is exemplified by the quote below from a metropolitan palliative care doctor who had set up telehealth services to serve a rural area that had no local specialist palliative medicine:

I feel quite strongly that people outside of metro Adelaide should, we should at least try for people to have the same access as those who live in Metro Adelaide. (palliative care doctor, P8)

Some participants, although not all, saw the potential for virtual consultations to address access issues of a practical and/or financial nature:

“[Patients have to]” pay to get the bus to come in, [or] don’t have a car, [or] don’t wanna pay for the petrol, [or] live an hour away from the hospital. (palliative care doctor, P5)

For another participant, telehealth opened the possibility of supporting health services in resource-poor settings where there was minimal access to palliative care:

we’ve always been wanting to try and help out (Pacific Islands). And, yeah, it’s fine, you can go to the Pacific Islands once a year, but if you could go there once a week virtually, that might make a bigger difference. (palliative care doctor P2)

The same doctor situated the use of telehealth services within a broader healthcare equity and socioeconomic context. From their perspective deprivation was not a limiting factor in being able to access a smart device and therefore telehealth:

it’s just that no matter how poor you are or how deprived you are in South Auckland, we’re a very mixed bag in terms of socioeconomic diversity, as you know. And most families will have access to a smart device of some sort because if you don’t have a smart device, be it the simplest smart phone, you’re just not connected with the whole world. And so there’s usually some point of access for most families. (palliative care doctor, P2)

On the other hand, compounding social and practical factors could contribute to the limited use of telehealth. The quote by a nurse below highlights how access to a device did not in itself make telehealth feasible:

There’s only three devices in the house with 11 people, kids trying to learn at school, mum’s trying to keep in touch to manage her son’s health needs, dad’s trying to work, and it just doesn’t work. It’s impossible. (nurse specialist, P6)

For this same nurse, the tension between the potential of telehealth to promote access and equity and at the same time contribute to inequity was a fine one as she recalls the stark contrast between sessions at an education day:

Where at the beginning of the day they [presenters] were talking about all the fantastic telehealth and how it was going to change the world and wonderful, and then at the end of the day we had the child Commissioner talking about houses where the windows are broken and they don’t have blankets. And I thought, that’s what we have to remember; not everybody has got the technology that they need to be able to access (nurse specialist, P6).

Communicating virtually through interpreters created further challenges to providing clinical care via telehealth. When services either weren’t available or the limitations of communicating via a screen were difficult for either party, patients could be lost in the system as a physiotherapist explained:

I think particularly when there was a language barrier, that was big. So not necessarily different backgrounds, but it was the English-speaking that was the big thing. So Self Direct does have the capacity to link with an interpreter but we weren’t ever able to coordinate that happening. Once I had a patient where the daughter would be present and help with interpreting, but again, that was only if they lived together because you couldn’t visit homes. I found that that was, some people dropped off because that was too hard. (physiotherapist, P11)

While study interview questions brought considerations about equity to the fore for some participants, practical considerations took precedence over sociocultural matters for the participant below:

I hadn’t really given it a lot of thought, to be honest (an optimal model for Māori). For me, it really is about what is acceptable and what a person can manage technically, as well. You know, if you don’t have the device, you can’t use it. And, you know, if you haven’t got the right skills. (Nurse Practitioner, P1)

Clinicians expressed particular challenges of virtual consultations in the context of caring for older people with cognitive impairment and hearing difficulties. Sometimes telehealth was deemed inappropriate in this context. For the clinician below, telehealth had the potential to further exacerbate cognitive issues:

I think someone who is, has cognitive impairment or who is suffering from delirium, the risk would be, it could make it even worse. You know, they’re seeing, talking to someone on a TV screen, they might be having delusions or hearing voices and having hallucinations. And then they are hearing a voice who is disembodied. They are talking to someone who’s not there. And so the risk of worsening their confusion would be there. (palliative care doctor, P2)

Conversely, however, the same participant reflected how even in this context telehealth had potential given the wider normalisation of ‘doing business’ virtually:

But I think it [telehealth] would still be worth trying and now, of course, most people in our population are more used to talking through screens, having virtual doctor visits, virtual talks with their insurance broker, virtual talks with their family. (palliative care doctor, P2)

While acknowledging the pragmatic and potential benefits of telehealth, clinicians emphasised the importance of retaining in-person consultations as the mainstay of palliative care. They emphasised the importance of context and individualising care for the person, family and situation as the nurse below conveyed:

We did have a guy with an intellectual disability that came on (the service) and died very quickly. So I was called out to him on a weekend and he was really miserable, poor soul. So getting him set up and trying to do symptom management and things with him. It was better to be face-to-face because he couldn’t [communicate via telehealth], you know, although his family were his advocates around that, but he was deteriorating very quickly. (nurse practitioner, P1)

### Patient safety and quality-complexities of clinical work

The rapid uptake of telehealth during the pandemic highlighted how important emotional connection and sensory aspects are for patient safety and quality of care. This included the risks associated with remote assessment as well as concerns about privacy and confidentiality. Participants articulated how they had to be more observant and focused during telehealth calls to ‘pick up’ on and interpret visual cues and other non-verbal cues. For instance, the quote by a Nurse Practitioner below shows how the absence of sight and touch made diagnosing dying more difficult:

the gentleman was dying, but, you know, it’s like I can’t see mottling [of the skin] on telehealth you know? But, when I see him in person I can look at, you know, look at his legs and I can feel his legs and I can, you know….I just have a much better sense. It’s kind of a full sensory thing rather than just somebody jiggling a phone in front of somebody. (nurse practitioner, P4)

Participants highlighted how being unable to fully assess the material environment and/or perform a physical assessment was not only a limitation of telehealth, but a potential safety risk. The nurse’s quote below highlights their concern that not being able to carry out a physical assessment could result in wrong diagnoses and subsequent mismanagement of symptoms:

one of the risks that I found talking on the phone and with the Zoom was the risk of missing stuff. Because you could not do physical assessment and you, god, all you have to do is a blood pressure and a pulse and the pulse is 140 and you’ve learnt something. But you do not pick that up on the phone necessarily, unless the person says my heart’s racing. But people can have it [heart failure] and not know it, just feel like they’re puffy and they’ve already got heart failure. So it can actually lead to assumptions I think and there’s a risk of wrong diagnoses and patients actually suffering because of that. (nurse practitioner, P1)

The aforementioned limitations of telehealth meant that clinicians often felt unable to ‘pick up’ on aspects of the person’s environment that would usually contribute to their overall assessment and subsequent clinical decision-making. The quote below shows how a home visit can provide critical information for safe prescribing of medications:

I think sometimes you look on paper and think ‘this is what I want to do’ with the medicines. And then you would go to the home and be like, ‘never in a million years is this gonna work’. This person’s not gonna manage to coordinate this regime of drugs I had in my head. What we have to do is strip everything back, keep it simple (palliative care doctor, P5).

Further, participants conveyed how clinical decision-making including prescribing of medications was inseparable from relational care of and with patients and families as the quote below shows:

Because it’s not really just about the drugs that we prescribe and the plan that we make. A bit of what we do is about the talking and the relationship and that, itself, is a bit of a medicine really, isn’t it, for the person (nurse practitioner, P10).

The paediatric palliative care clinicians we spoke with conveyed how video consults with children could pose additional challenges. These clinicians shared how their usual strategies for engaging children in the consultation, by, for instance, play activities, were often curtailed in the online setting:

There might be two of us [clinicians] with a family and one of you might go off and sit with the two-year-old sibling and play blocks and talk with them or sit with the six-year-old patient. And, you know, they ask questions and talk about what’s happening and, you know and play. So I think we use touch, yes, but I think play is a big part of it too. (nurse specialist, P6)

The importance given to relational aspects of care meant that safety concerns extended to matters of cultural safety. The same specialist paediatric nurse, making explicit her reference to a Māori family and cognisant of the concept of whanaungatanga (where relationships require trust, authenticity and are reciprocal) contrasts the overt nature of a telehealth consult with what could be a much more discreet and sensitive conversation in person with the father of a patient.

He [the patient’s father] was usually a very quiet, considered Māori man who was very engaging usually. But I think it would’ve been the person we’d have had that quiet, informal conversation with walking out to the car rather than overtly. And when we did Zooms, it tended to be on mum’s phone and mum would hold the phone and talk and he would have an opportunity to speak but I guess it wasn’t the same as if we were in the house, in the same room together. (nurse specialist, P6).

In addition, participants were conscious of potential privacy and confidentiality issues in the context of telehealth including the security of patient information and use of devices. As one participant noted:

There’s also situations where I don’t know what phone they (patient) were using, or whose phone they were using, but, you know, they were using a phone and so privacy wise not up to par. (Nurse Practitioner, P4)

Further, since the clinicians’ field of view was restricted, it was not always possible for them to determine if the patient was alone or accompanied by a caregiver or a family member for the consultation call. To address this, some participants explicitly enquired during the call if “there [is] anybody else and [if] are we on speaker”. This also gave rise to concerns that patients might conceal details about their condition in the presence of a caregiver, impacting clinical care and recommendations.

The absence of appropriate devices and suitable workstations for clinicians to conduct virtual consultations not only made it difficult for them to work but also raised concerns about patient privacy. Open plan offices did not offer enough privacy for video consultations, even offices with thin walls made clinicians conscious: *“that people in the offices could probably … hear some of it*,* if not all of what they were saying”.* The quote by a specialist palliative care doctor below illustrates how communication challenges during virtual family meetings can contribute to misunderstandings. In this instance, the clinical team missed non-verbal cues from a family member, leading to a significant miscommunication between the clinical teams and the family.

It was a story about a family meeting and, you know the traditional family member going like this behind, [gestures a pulling face]. And this was missed by another team [member] and that led to a huge misunderstanding and a formal complaint. Because they [family] didn’t believe that the clinicians had actually properly addressed all the other family issues or heard [their concerns], or completely assessed the problem. (palliative care doctor, P 19)

The aforementioned safety concerns occurred in the context of a significant additional workload against the backdrop of the pandemic. This ‘articulation’ work was often ‘unseen’ and included learning to use new technology and transitioning their clinical and interpersonal skills to a new modality of delivering service. Further, informal expectations were placed on clinicians who were more adept at using technology to help their colleagues and patients with navigating these systems. This work often went unseen, placing clinicians under additional pressure as a physiotherapist conveyed:

And we found, as therapists, it was really draining to be on the screen all day and lots of the patients cry because of lockdown and cancer and all the complexities around that. So it was pretty emotionally draining for the staff. (physiotherapist, P11)

### Tele-caring, connection and creativity

As clinicians became more experienced and comfortable with telehealth, they often developed creative strategies and approaches to enhance their assessments and care delivery. When unable to visit a person, either because of distance or COVID restrictions, clinicians would often enlist the help of those who were physically present to assist in their assessment. Sometimes, this was the patient themselves, as this senior dietician conveyed:

And you rate those [patients] based on how malnourished or how much muscle fat they’ve lost. Now, that’s obviously the part of my assessment that I can’t do (virtually), it’s a validated tool. So I’ve had to adapt my practice to go, “okay, can you just show me this, can you lift up this and show me?” And I’ve not had a patient complain. They’re all like, “Yeah, yeah, can you see this?” And I’m like, and or I’ll ask them, “can you feel, is there is a bit of a divot here or is that high or is that low?” (dietician, P16).

This could mean asking patients to perform specific tasks to inform the virtual clinical assessment that might otherwise be incidental had the clinician been performing the assessment in person, as this nurse explained:

It’s a bit like when you walk into a room and you see someone for the first time, you know whether they look sick or whether they don’t. You know, whether they look in pain. And so things like getting the patient to stand up and move and show me how, what their gait’s like. (nurse practitioner, P8)

Family caregivers of patients also took on an extended role in clinical assessment. Clinicians frequently guided both patients and families through various tasks, including physical assessment:

So, it’s actually amazing how you can talk through some basic physical assessment with families, even when they’re not health professionals. (nurse practitioner, P13)

Specialist clinicians would also enlist the support of health care professionals who were able to be physically present; “patients actually engage in it and so does the nurse … and I’ll guide them. … so it’s pretty collaborative.” (palliative care doctor, P8). The quote by a doctor below highlights how assessment is a collaborative effort reliant on both a patient’s caregiver and the district nurse to make an assessment of whether a person should stay at home or be admitted to hospice or hospital:

Oh, he’s really sleepy and drowsy today. And I was like, you know, you have to then rely on her [patient’s wife] and asking “oh well, is he hallucinating? Have you noticed any twitches?” And all you want to do is get in your car and drive to the house and have a look and then you’d feel like, okay, I know what’s happening, you [patient] need to come in (to hospital) or we can keep you at home. And so then you’d have to speak to the district nurse and say, what do you see? They [district nurse] were sort of your eyes on the ground, like, what do you see, what does it look like? (palliative care doctor, P5)

Some participants, although not all, contrasted the challenge of building rapport via telehealth with how they made connections face-to-face. For instance, the nurse below expressed how they drew from the Māori concept of whakawhanaungatanga (the practice of building relationships and connections with others) to try and build connections through informal conversations about the person’s environment and/or artefacts.

Because normally in the aged care space the first thing I do when I go into the room, and in the hospice space. And when you go into home is, you know, try to find some way to connect the whole whakawhanaungatanga thing and connect with somebody. Whether that be something that you notice in their home or in aged care a photo that’s near their bed or what they’re reading or something, you know? (nurse practitioner, P13)

Further, participants, including those with limited previous experience of using telehealth, often expressed their surprise at how rapport was possible despite the limitations and challenges posed by technology as the clinician below conveyed:

The warmth and the caring, yeah despite never having met me and that was only after one Zoom. So, you know I thought ‘goodness, you know this is really possible to have these connections’. And to establish rapport, and to get some of this work done that will make a difference. (paediatric palliative care doctor, P12)

The kind of connections and care participants talked about could sometimes challenge their own assumptions and take clinicians by surprise. For instance, a nurse practitioner conveyed how virtually connecting family members, unable to access aged care facilities due to COVID restrictions inadvertently promoted person-centred care for people with dementia:

And several of them [people with dementia], you put the tablet in front of them with Zoom with their loved one on it and it’s like all of a sudden they engaged a whole lot more. And I’m not sure what that was, you know, like it was, and it was striking. You know, like they just wouldn’t be engaging and then you put that [tablet] in front of them and it’s like all of a sudden they kind of wake up. (nurse practitioner, P4)

## Discussion

Our findings, similar to those of previous studies [27] showed that, for the most part, clinicians reported that the adoption of telehealth could extend the reach of palliative care by optimising time and service capacity while minimising the need for travel. Nevertheless, not all sub-groups of patients necessarily derive benefit. Our research highlighted that patients were often expected to rely on informal caregivers for support with telehealth. This support extended beyond assistance with the technology itself to include physical assessment by becoming the eyes, ears and hands of clinicians ‘on the ground’. This has the potential to place an additional burden on informal caregivers who are expected to take on clinical as well as caring roles and tasks as part of the wider healthcare system drive for homecare [28]. Furthermore, as one participant described, telehealth can be a “two-edged sword”. That is, there was a risk of telehealth contributing to the ‘inverse care law’ [29] and the ‘digital divide’ (the gap between people who can access and use digital technology and those who cannot). In the Australian context, data have shown that this divide is substantial and continues to widen for some groups, especially older people [30]. In other words, rather than promoting access and equity of palliative care, telehealth could, if not used appropriately, risk further marginalising those who already have difficulty accessing specialist services. As the work of Frydmen et al. has also highlighted [31], this included but was not limited to: people who do not have access to the internet or devices or who are unfamiliar or uncomfortable with software and technology; people with communication difficulties; people with intellectual disability; people with cognitive impairment, and those whose first language was not English. Participants in our study rarely made explicit mention of particular needs, opportunities or barriers to telehealth specifically for First Nations people despite this being an interview question. While telehealth had the potential to extend palliative care services to underserved rural patients, limited reliable internet access impacted the capacity to do so. A recent study from the USA exploring palliative care providers’ perspectives of telehealth and underserved populations highlighted that this issue is not unique to Australia and Aotearoa New Zealand [32]. Our findings highlight that when integrating telehealth into the care of people with palliative care needs, there is a need to account not only for the challenges faced by different groups of people but also for individual patients and families, their context and individual circumstances.

Our findings are consistent with those of previous research findings. That is, the rapid shift to telehealth during COVID-19 placed expectations on clinicians to transition their clinical and interpersonal skills to a different modality of service delivery with minimal training and preparation time [33]. They were largely left to identify and evaluate their own and their colleagues’ proficiencies in using telehealth. The ‘normative expectation’ that clinicians would simply ‘get on with it’ rarely took account of the ways in which telehealth changed the nature of the consultation. For example, clinicians found play activities difficult to replicate online despite being considered integral to developing relationships with children [34]. While telehealth was found to be useful for maintaining regular contact with patients under pandemic circumstances, its effective implementation required significant and often invisible work by informal caregivers as well as clinicians who had to familiarise themselves with technology and devise ways to effectively build interpersonal relationships online. Clinicians, similarly to those in English et al.’s study, reported varying capacity in being able to build rapport with patients and families through the digital medium [33].

Our findings show that clinicians frequently found ways of problem-solving and adapting their skills using their existing expertise. They often developed creative strategies and approaches to optimise care for patients and families and with other clinicians. Working within the constraints of communicating through computer monitors and in shared office spaces, they sometimes struggled to gain a comprehensive picture of patient and family situations and associated needs and/or to ensure patient privacy was maintained. These challenges, combined with limited access to non-verbal cues and few opportunities for informal interactions, resulted in fears that consultations could become narrowly transactional and lead to compromised patient safety and quality. While affirming pragmatic gains enabled by telehealth, clinicians cautioned against considering telehealth services as a replacement, rather than a supplement, for in-person visits and consultations. This finding is aligned with those of previous studies [2]. When discussing the impact of telehealth, clinicians, like their US counterparts in a similar study [32] were, for the most part, positive about its potential to extend the reach of their services while streamlining their work and enabling them to optimise the use of their time to make palliative care available for more patients. Telehealth also enabled family members as well as non-specialist clinicians to be present with patients virtually, thereby widening the community of care around the patient and facilitating continuity of care.

In addition to changing the dynamics of clinician-patient interactions, our study shows that the adoption of telehealth could alter the work setting, causing strain for clinicians and impacting the quality of care for patients. The streamlining of work enabled by telehealth and the increased number of consultations in a day were seen as benefits, but these shifts also led to clinicians experiencing “screen fatigue” and feelings of stress when they did not get adequate “down-time” between consults to process emotionally difficult conversations with patients. While the more obvious limitations with virtual consults – the inability to perform physical assessments and complex procedures – are relatively well understood, changes in the more intangible, affective elements of clinician–patient relationships and the impact of altered work settings on clinicians are not yet fully understood. As telehealth becomes integral to palliative care models, and clinical care in general, more work is needed to develop service models and sustainable work patterns that ensure safety and quality of care for patients and families. This includes cultural safety and avoiding further marginalisation of those who already have difficulty accessing palliative care.

### Limitations

This research is based on interviews conducted with palliative care clinicians from Aotearoa NZ and Australia. Thus, our findings might not be transferable across different geographical and cultural settings. Our study would have been strengthened by a greater diversity of clinician voices, especially those from Australia and those from rural and regional areas. Māori have unique palliative and end-of-life care needs not always met by current health services [17]. This inequity was exacerbated during Covid-19 [35]. Likewise, Aboriginal and Torres Strait Islander peoples are under-represented in palliative care and there is a paucity of research on the cultural acceptability of telehealth for use by Aboriginal and Torres Strait Islanders [36]. Despite the initial aim of our the study to include the impact of telehealth for First Nations people, this aim was not achieved. A significant limitation of our study was that despite having a Māori researcher on our team at its inception, the multiple competing demands on First Nations researchers compounded by the pandemic resulted in no First Nations people’s voice in data collection and analysis. Further, none of the participants identified as Māori or Aboriginal and Torres Strait Islander. Nevertheless, participants worked in areas that served First Nations people. A further limitation was that most nurses in our study were senior nurses (nurse specialists or nurse practitioners). Less experienced nurses may have a very different perspective. While patients’ and caregivers’ experiences of navigating telehealth were outside the scope of this study, further research with diverse groups of patients and families is required to generate insights into the individual competencies, organisational structures and models of care as well as policy changes that would be required for safe and quality palliative care via telehealth.

## Conclusions

The widespread adoption of telehealth under pandemic conditions has provided the opportunity for significant learning about the feasibility and efficacy of telehealth and its ongoing use in palliative care, particularly for underserved groups. That participants, for the most part, did not convey specific barriers to telehealth for First Nations people does not mean there are none especially given our study limitations. Taking into account evidence showing the difficulties First Nations People already experience accessing the current health system as well as during COVID-19, designing culturally appropriate telehealth services with and for Māori and Aboriginal and Torres Strait Islander peoples could have positive outcomes in the delivery of palliative care services [23, 37]. There is a critical need to understand patient, family and whānau experiences for those underserved by palliative care, along with clinical outcomes during the process of introducing telehealth models in palliative care contexts to ensure culturally safe telehealth and to avoid widening the “digital divide”. A key finding was that as well as technological training and support for clinicians and patients, there is a need for support and educational preparation for its use, including: clinical assessment (considering when and for whom telehealth is appropriate); safe and quality care via telehealth, developing relationships and rapport via telehealth and managing privacy and ethical issues. At the organisational level, providing appropriate work settings for conducting telehealth consultations while maintaining patient privacy and adopting work patterns that minimise the burden on clinicians and provide them with adequate opportunities for interaction and knowledge-sharing is important to developing a sustainable model for telehealth. Clear organisational guidelines around patient selection and service delivery expectations might guide the selection of telehealth-appropriate patients, which could potentially free up more time for patients and families who are not suitable for telehealth. This might also help alleviate distress and uncertainty for clinicians. Further, understanding the needs of different patient sub-groups and developing telehealth consultation practices while also acknowledging inequities in technological access is important to ensure that existing inequalities in access to palliative care are not exacerbated.

## Data Availability

Deidentified study data is available from the corresponding author upon reasonable request.
